# Acute Periostitis

**Published:** 1852-07

**Authors:** J. H. M'Quillen

**Affiliations:** Dentist.


### ARTICLE XIII.

*Acute Periostitis.* By J. H. M'Quillen, M. D., Dentist. Read before the Pennsylvania Association of Dental Surgeons, and published at their request.

*Acute periostitis*, or inflammation of the membranes investing the alveola and roots of the teeth, is an affection of frequent occurrence; and its local effects are sometimes attended with serious consequences. The various diseases of the antrum, in the majority of cases, can be traced to this as the exciting cause; also, spina ventosa, osteo sarcoma (usually considered a malignant disease,) necrosis, and exfoliation, in either maxilla, may nearly always claim it as a common parent. Following these, permanent deformity of the "face divine" has resulted as a sequence.

*Causes.*—Destruction of the dental pulp may act as an exciting cause, whether resulting from simple exposure to variations of temperature; acrid secretions of the mouth; compression of the pulp, from lodgment of foreign bodies in the cavity of decay, as portions of food, or the insertion of a plug; and, lastly, the exhibition of a corrosive agent, at the hands of a dentist. This, however, is a rare excitant, when the treatment is properly conducted. The destruction of the pulp, not followed by its removal, and plugging the dental canal, but merely filling the cavity of decay, is, in the majority of cases, soon followed by the appearance of this affection. The state of utero-gestation appears to be a condition of the system particularly prone to the development of this affection (when there is a predisposition that way, owing to the pressure of diseased roots and teeth.) This is easily accounted for, by the cerebral determination so characteristic of this state. The attention of the writer was directed to this circumstance several years ago, by Professor J. D. White; his own experience since that time, has confirmed the correctness of the observation. It appears, however, to have escaped the attention of dental writers, as none have mentioned it in their productions. And yet, the knowledge of this, is a matter of moment, as by well-timed and judicious operation, much suffering could be spared those who suffer most.

Like inflammation in other parts of the body, this is divisible into three stages, viz. simple vascular excitement, active congestion, and true inflammation.

The first stage, which is simply a hyperemic condition of the capillary vessels of the membrane, the circulation through the part proceeding, at the same time, with unwonted velocity, merely occasions a slight amount of tenderness, which is increased by striking sharply on the affected tooth with the handle of an instrument. A sense of relief is obtained, by gradual and continued pressure upon the tooth; which, however, on being removed, is followed by a recurrence of the tenderness. At this point, without any attention from the practitioner, the vessels, by unloading themselves, and decreasing to their natural calibre, the part will return to its normal condition. Should the action not cease here, but proceed, *active congestion* is established: the capillaries become more and more distended, until distension is no longer possible, owing to the confined locality of the tissue; exudation of the liquor sanguinis take place to a slight extent; the most excruciating pain is now experienced, and along with it, intolerance of the slightest pressure. On this account, closure of the teeth is avoided, as it but aggravates the evil. The tooth seems longer and looser than its neighbors—and such is really the case; for, owing to the distension of the blood-vessels of the membrane, the root is forced slightly out of its socket. The pain is sometimes intermittent in its character, but in the majority of cases is continued; an increase of it is, however, experienced by nearly all towards evening. This is attributable to the febril exacerbations. By proper and active treatment, the action may possibly be arrested even here. This, not succeeding, or being attempted too late, it advances to *true inflammation*. Effusion of plasma takes place to a greater extent than in the previous stage; stagnation of the circulation through the part occurs; in the surrounding tissue on the contrary, it is going on with increased activity. The pain is lancinating in its character, owing to the efforts of the blood on the cardiac side to overcome the obstructing mass; the gums are very much swollen, excessively tender, and unnaturally red; the side of the face on which the affected tooth is situated, becomes edematous, and increases to an unnatural size. After this has continued for a shorter or longer period *suppuration* takes place, by *disintegration of the effused plasma.* The abscess is generally situated at the apex of the root. It usually makes its escape from the socket by the opening effected through the thin parieties of the alveolus, on the buccal side, though sometimes the lingual. A place of exit being thus procured, the abscess advances through the gum, and arriving at the surface, evacuates its contents into the mouth. Under favorable circumstances, this is followed by a cessation of pain, and a restoration of the part involved to a healthy condition. Such a favorable termination is not always to be anticipated. The abscess may open into the antrum, or even when it has made its way to the gum, it may not approach the surface, but burrow, and thus the consequences alluded to, may result, if an opening is not made quickly and boldly by the lancet.

*Treatment.*—Should the tooth be a valuable one, with the prospect of future usefulness, every effort should be made to save it. The practitioner should always bear in mind, and impress upon his patient, the fact, that even after the part has been restored to a healthy condition, that it is liable, at any moment, to take an inflammatory action. As it is an axiom in surgery, that a part which once has been truly inflamed, leaves that part much more susceptible to the action of exciting causes, and hence is more liable than healthy tissue to become the seat of subsequent disease. Having decided to save the tooth, the treatment in the first and second stages is somewhat analogous, the only difference consisting in its being more active in the latter than in the former. Slight scarification of the gum, cold or warm water, (as is most comfortable to the patient,) held in the mouth, may suffice for the first; but the application of two or three leeches to the gum will be imperatively demanded, to prevent the establishment of the inflammatory acmé in the second. Now, the idea of having a leech placed in the mouth, is repugnant to the feelings of all, and particularly the over-sensitive. It therefore affords the writer much pleasure to invite your attention to a recent invention of a French gentleman, which is called an artificial leech. It consists of a glass tube or cylinder, with a piston fitting accurately, and attached, by means of India rubber, to a piece of horn, situated at the upper orifice of the tube; the horn is perforated through its centre, to admit a small steel rod, by which the piston is forced down to the lower orifice. Applying it now to a gum recently scarified, and removing the rod, the India rubber contracts and draws the piston to its former position; a vacum is thus formed, into which the blood flows freely, owing to the removal of the atmospheric pressure, and consequent distension of the blood-vessels. As much blood can thus be removed as the practitioner may deem necessary. Having used it, and found it to answer the purposes indicated, I can vouch for its utility.

As a case in point, Miss T——, a lady of an excessively nervous temperament, called upon me to have a lateral incisor of the right superior maxillary plugged. In removing the decay, the pulp was found to be exposed. Forthwith, the arsenical paste was applied, to destroy its sensibility. At the expiration of eighteen hours, the pulp was removed; there was a slight hemorrhage from the upper part of the dental canal, but no painful sensation was experienced by the patient, either from the introduction of a fine and flexible probe, passed up the canal as far as practicable, or upon striking the tooth sharply with the handle of an instrument. Introducing a pledget of cotton into the pulp cavity, and packing it firmly, so as to arrest the slight amount of hemorrhage—thus preventing the imbibition of the coloring matter of the blood into the tubuli, and consequent discoloration—the patient was directed to call the next day. On the following day, the bleeding had ceased; the dental canal and cavity of decay were then plugged with gold foil. Owing to the temperament of the patient, and the fact of her being under treatment for a neuralgic affection of the eyes, I still, notwithstanding the absence of the slightest sensibility, apprehended the establishment of some irritation, and therefore requested to be informed, if pain supervened, as soon as possible. At the expiration of four or five days, the patient called and told me that the night before, she had been attacked with a slight pain, which had augmented in severity until it had become almost insupportable. The artificial leech was applied, and from one to one and a half ounces of blood removed. The application of cold water to the part, to be continued for some time, was then advised. This plan was followed by the happiest effects.

When, however, the third stage is established—indicated by the puffy condition of the gums and face—*resolution* is no longer practicable; depletion, therefore, is not admissible, as it retards the suppurative stage—the indication being to promote this as speedily as possible, and relieve the sufferer of the intolerable pain. Emollient poultices applied to the gum, as roasted figs, or flaxseed enveloped in a gause sack, will be found very serviceable in this stage. The domestic practice of enveloping the entire side of the face in a huge poultice, cannot be too highly deprecated, as it has a tendency to induce the abscess to point at the surface of the cheek, and possibly open through it. Many persons carry a scar there, as an evidence of this injudicious custom. Two or three days days will generally suffice for the formation of pus; therefore, a practitioner who will allow his patient to go an entire week, without attempting to effect its evacuation by the lancet, is culpable in the extreme.

On Friday, October 17th, an Irish laborer presented himself at the office, with the right side of his face very much swollen, and complaining of considerable pain. Upon requesting him to open his mouth, I found that he could only separate the jaws sufficiently to introduce the little finger between the teeth. Requesting a history of the case, the following was given: A week previous, an unsuccessful attempt was made to extract the dentes sapientiæ of the inferior maxillary, right side. During that night, the pain became lancinating in its character, and on the following morning, the face presented the edematous appearance described above. Thus a whole week had been passed, of intolerable suffering—the person, at the same time, being incapacitated for the performance of his daily avocation. It was plainly perceptible that the abscess was pointing towards the surface of the cheek. As no time was to be lost, a free incision was made internally with the lancet, which was followed by a profuse discharge of pus. A small piece of cotton was then placed in the wound, to keep its lips patulous, and also to act as a tent, to drain off the pus that otherwise might accumulate in the abscess, and necessitate the use of the lancet again. The patient was requested to call in a few days, to have the roots removed. At the time specified, they were extracted without any difficulty. The pain had disappeared, and the face had resumed its natural expression. In conclusion, the writer would cite a case of malpractice, that came under his notice about five years ago, whilst in the office of his preceptor. The patient, in this case, was by occupation a cab-driver, and therefore exposed to all the inclemencies of weather and variations of temperature. The history of the case was as follows: A second molar of the inferior maxillary, left side, was attacked with periostitis, and resulted in the formation of an abscess. Owing to the application of one of the huge poultices alluded to, it pointed externally. At this crisis, he called upon a druggist, who lanced it *externally*, and in the course of a few days, applied a plaster, to promote the healing, and closure of the wound. This, however, proved futile, and on the contrary, a fistula was formed, through which the pus exuded. This continued four or five weeks, when the patient was advised to call upon my preceptor. Looking upon the tooth as the cause of all the mischief, he at once removed it; this was followed by a disappearance of the fistula, but a deep pit was left as an evidence of the impropriety of lancing externally.

Perfectly conscious of numerous imperfections, and want of style and arrangement, and the possible adoption of erroneous views, these remarks are submitted to you. If the latter is the case, now is the time for correction ; and the writer will be but too happy to acknowledge himself in error, if those errors are pointed out, and correct views substituted in their place. He therefore hopes that this may draw forth the views of the members present.—*Dental News Letter.*

